# Low‐Power Optoelectronic Synaptic Transistors with Multimodal Neuromorphic Computation and Retinal‐Inspired Multiband Optical Binary Communication

**DOI:** 10.1002/smsc.202400511

**Published:** 2025-01-21

**Authors:** Bo Huang, Linfeng Lan, Jiayi Pan, Fuzheng Qi, Jing Li, Churou Wang, Yaping Li, Dechun Zeng, Jiale Huang, Jintao Xu, Junbiao Peng

**Affiliations:** ^1^ State Key Laboratory of Luminescent Materials and Devices South China University of Technology Wushan Road 381 Guangzhou 510640 P. R. China

**Keywords:** audiovisual fusion effects, dextran films, multiband optical communications, optoelectronic synaptic transistors, visual adaptations

## Abstract

Biomimetic neuromorphic optoelectronics exude tempting attraction in multimodal interaction and visual applications because of their capability of integrating sensing, memorizing, and processing in a single device. Herein, a natural dextran film that is intrinsically green and transparent is employed as the dielectric of the optoelectronic synaptic transistors (OSTs). The resulting dextran‐OSTs that operate at an ultralow energy consumption (15.89 aJ) exhibit multimodal neuromorphic computation ability with excellent synaptic plasticity, including pair‐pulse facilitation (PPF, as high as 494%), spike voltage/frequency/duration/number‐dependent plasticity, and a high recognition accuracy of 89.95% by handwritten digital datasets. Furthermore, the device exhibits visual self‐adaptation ability and audiovisual fusion effect, showcasing the immense potential in self‐adaptation and synergy sensing. More importantly, the dextran‐OSTs can significantly advance the capabilities of binary optical information processing and memorizing. This demonstrates the great advantages of dextran‐OSTs in multimodal neuromorphic computation, visual self‐adaptation, synergy sensing, and multiband optical communication.

## Graphical abstract

A natural dextran film that is intrinsically green and transparent is employed as the dielectric for the optoelectronic synaptic transistors (OSTs) with the function of sensing, memorizing, and processing. The multimodal neuromorphic computation, visual adaptation ability, and retina‐inspired multiband optical binary (0 and 1) communication can be correspondingly mimicked by the single dextran‐OSTs.

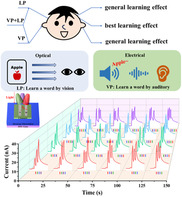

## 1 Introduction

Optoelectronic synaptic transistors (OSTs) to integrally mimic multimodal perception, computation, and memorization have provoked intensive interest in the next‐generation artificial intelligence (AI) for potential applications in augmented reality (AR), human–computer interaction (HCI), neuromorphic processing, etc.^[^ ^1^, ^2^, ^3^ ^]^ The optical/electrical plasticity and energy‐efficient computing‐in‐memory (CiM) ability make OSTs one of the hotspots in current research.^[^ ^4^, ^5^, ^6^, ^7^, ^8^, ^9^ ^]^

The existing efforts on mimicking visual adaptation functions have been trapped in complicated hardware and algorithms that typically reduce operating efficiency, and the new generation of artificial visual systems should have a simpler structure and lesser logic operations that can automatically adjust the response according to different environments.^[^ ^7^ ^]^ Besides, audiovisual fusion and Pavlov's dog effect are typical representatives of synergy sensing, also constrained by hardware facilities.^[^ ^10^, ^11^ ^]^ The OSTs are one of the best candidates for visual adaptation and synergy sensing, which can respond to the electrical gate voltage (*V*_G_) pulse signal and the light pulse signal to simulate the human auditory and visual systems. Besides, the feedback of OSTs to different light wavelengths improves the precision, bandwidth, and speed of the optical communication and further becomes the key factor for reservoir computing (RC).

Oxide semiconductors represented by InGaZnO_4_ (IGZO) are generally excellent optoelectronic responsive materials that have the advantages of high mobility, low off current, low energy consumption, and low cost.^[^ ^12^, ^13^, ^14^ ^]^ The conductance can be largely modulated by electrical/optical stimulations. In addition, the carrier recombination time is long for IGZO because of the large lattice relaxation for the ionization of the oxygen vacancies, which is good for synaptic plasticity and persistent photoconductivity (PPC).^[^ ^15^, ^16^, ^17^, ^18^ ^]^

Recently, natural and green materials become more and more attractive in the advancing neuromorphic devices.^[^ ^19^ ^]^ Actually, natural polymer is one of the most economical and convenient materials for the preparation of bionic devices. Dextran is an attractive natural biomaterial extracted from sucrose fermentation with the advantage of biodegradability, biocompatibility, and nontoxicity^[^ ^20^, ^21^ ^]^ Abundant protons (H^+^) and natural green/transparent characteristics make the dextran one of the best candidates for constructing synaptic transistors.

Here, a novel OST with multimodal neuromorphic computation and multiband optical communication was constructed using IGZO as the channel and dextran as the electrolyte. The OSTs exhibited excellent electrical performance at an ultralow operation voltage (≤1 V) with mobility (*μ*) up to 14.3 cm^2^ V^−1^ s^−1^. Furthermore, to construct electrical/optical multimodal neuromorphic computation, the optoelectronic plasticity has been investigated, including short/long‐term plasticity (STP/LTP), pair‐pulse facilitation (PPF, as high as 494%), spike voltage/frequency/duration/number‐dependent plasticity (SVDP/SFDP/SDDP/SNDP), and learning‐forgetting‐relearning feature. The device exhibited a high recognition accuracy of 89.95% by handwritten digital datasets. Based on the excellent optoelectronic plasticity, the visual adaptation and synergy sensing ability were successfully simulated. Finally, an optical communication system based on multiband light (*λ* = 620, 520, 465, and 395 nm) was successfully constructed. The OSTs can discriminate light with different wavelengths, achieving retina‐inspired multiband optical binary communication (retina‐inspired intelligent communication). It is for the first time to employ dextran as the gate dielectric for IGZO (or oxide) synaptic transistors. This research promotes the development of the emerging brain/retina‐inspired bionic devices that have the characteristics of multimodal interaction and optical information dissemination.

## 2 Results and Discussion

### 2.1 Characterization of the Dextran‐OSTs

**Figure 1:**
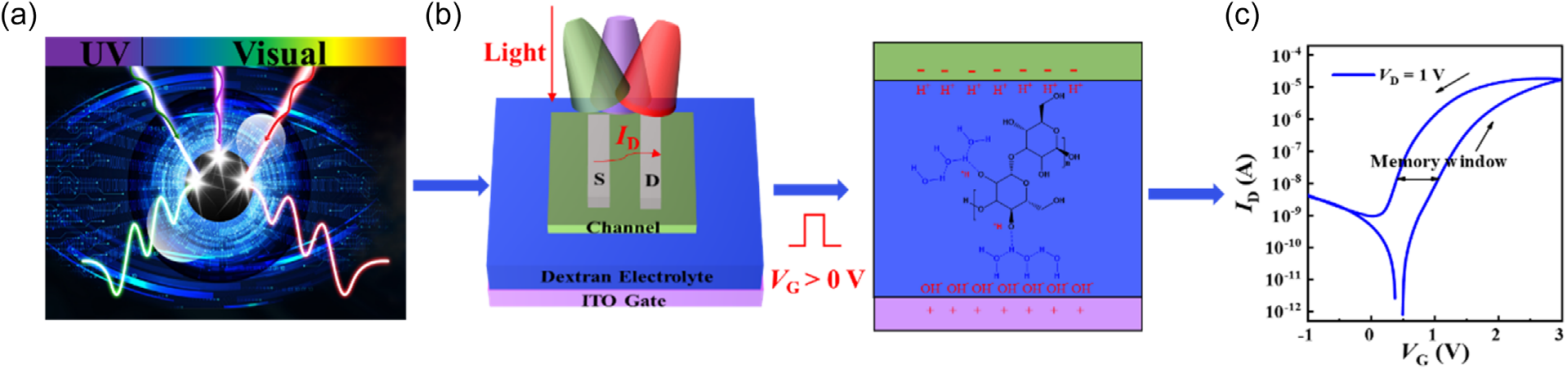
Schematic diagram and performance of the dextran‐OST. a) Schematic diagram of the retina‐inspired computation and multiband optical binary communication. b) 3D diagram of the dextran‐OST and the principle of optoelectronic regulation. c) Transfer characteristics of the dextran‐OST.

**Figure** **1**a,b shows the concept of the retina‐inspired neuromorphic computation/multiband optical binary (0 and 1) communication and the schematic structure of the dextran‐OSTs. UV‐vis light with different wavelengths was encoded as optical binary. The detailed fabrication process is illustrated in Figure S1, Supporting Information, and described in the Experimental Section. The dextran molecules contain a large number of hydroxyl groups that can absorb water from the surrounding environment. The protons (H^+^) in the film may originate from the dissociation of hydroxyl groups in the dextran molecule. The oxygen atoms dissociated in the dextran and the hydrogen atoms in the water molecule form a hydrogen bond network, in which protons migrate. When *V*_G_ > 0 V, the H^+^ in the dextran film moves rapidly to the dielectric/channel interface, forming an electric double layer (EDL) with large capacitance. The effects of the precursor concentration and dextran film thickness on the roughness, capacitance, transfer curve, and leakage current were investigated, as shown in Figure S2, Supporting Information. As the dextran thickness (*d*) increased from 60 to 340 nm, the surface roughness was almost unchanged (see Figure S2a, Supporting Information). However, a pronounced decrease in capacitance was observed (from 350 to 50 nF cm^−2^, Figure S2b, Supporting Information). Furthermore, the dextran‐OSTs exhibited relatively high leakage at lower thicknesses (*d* = 60, 145, and 160 nm), as shown in Figure S2c, Supporting Information. However, when *d* > 175 nm, there was a significant reduction in leakage current (≈1 nA @ *V*_G_ = 3 V). When *d* = 175 nm, the transfer curves exhibited a more distinct memory window, which is beneficial for simulating synaptic behaviors. With further increases in the thickness (*d* = 340 nm), the memory windows decreased, which may be attributed to the significant decrease in capacitance. As shown in Figure S3a,b, Supporting Information, the surface morphology of the optimized dextran film was characterized by atomic force microscopy (AFM) and scanning electron microscope (SEM). A smooth surface with a roughness of only 0.202 nm facilitates the formation of a good interface contact between the channel (IGZO) and the electrolyte (dextran dielectric layer), thereby enhancing the electrical performance of the device. As shown in Figure S3c, Supporting Information, the dextran film exhibits a transmittance exceeding 98%, rendering it highly advantageous for transparent electronic applications.

The output curve of the optimized dextran‐OST is shown in Figure S4, Supporting Information, indicating that the dextran‐OST is an enhancement mode transistor. The corresponding transfer curves are shown in Figure 1c (*V*_D_ = 1 V), Figure S5a, Supporting Information (*V*_D_ = 0.1 V), and Figure S5b, Supporting Information (*V*_D_ = 0.5 V). It exhibited excellent electrical performance at an ultralow operation voltage (≤1 V) with mobility up to 14.3 cm^2^ V^−1^ s^−1^, subthreshold swing (SS) of 241 mV dec^−1^ and on/off ratio of 10^5^.

### 2.2 Synaptic Behaviors under Electrical Stimulation

**Figure 2:**
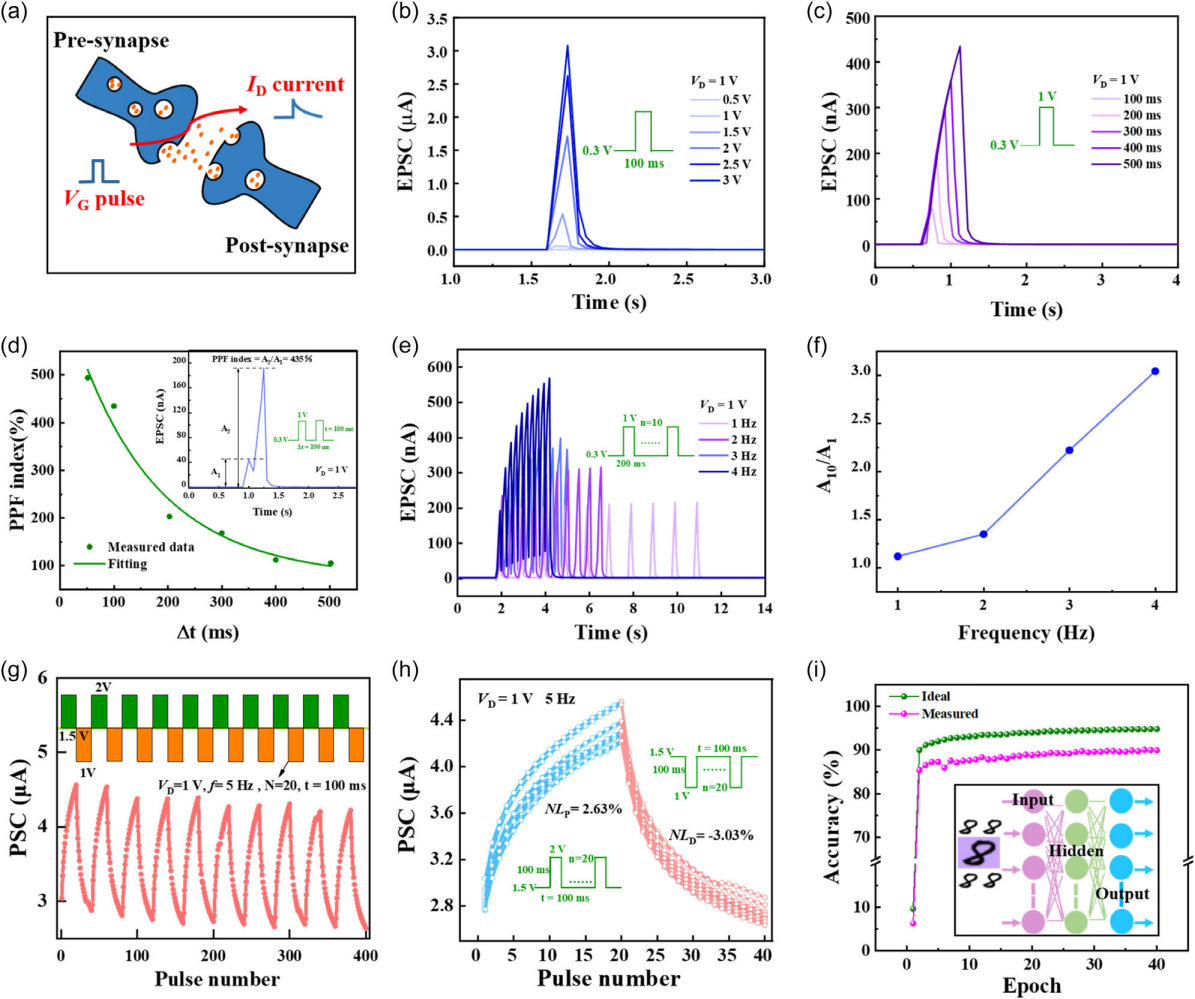
Principle of synapse and synaptic performance under electrical stimulation of the dextran‐OST. a) Schematic diagram of the principle of the synapse. b) Single presynaptic pulse‐induced EPSC with different voltage. c) Single presynaptic pulse‐induced EPSC with different pulse widths. d) PPF index with different Δ*t* (inset: two successive presynaptic pulses with *Δt* of 100 ms). e) The filter effect at different frequencies. f) *A*_10_/*A*_1_ at different frequencies. g) PSC under repeated successive multiple positive‐negative pulse groups. h) The average NL value of PSC. i) The recognition accuracy of 28 × 28 pixel handwritten digital images with training epochs measured (inset: MNIST dataset and neural network with the layers of three layers ANN).

**Figure** **2**a illustrates the working principle of the biological synapses. The efficient transmission and processing of pulse signals by synapses is the fundamental pathway for the brain to achieve cognitive and learning functions. To demonstrate synaptic plasticity under electrical stimulation, the excitatory postsynaptic current (EPSC) triggered by electrical pulses was investigated, which is reflected by drain current (*I*_D_). As shown in Figure 2b,c, EPSC increased with an increasing pulse level or pulse width. The response current (ReC), the memory current (MeC), and the memory degree (MeD) are defined as Equation (1)–(3), respectively^[^ ^5^ ^]^.

(1) $$\text{Response current }\left(\text{ReC}\right)=\left|I_{1}-I_{0}\right|$$

(2) $$\text{Memory current }\left(\text{MeC}\right)=\left|I_{2}-I_{0}\right|$$

(3) $$\text{Memory degree }\left(\text{MeD}\right)=\left|\left(I_{2}-I_{0}\right)/I_{0}\right|$$

where *I*_0_ represents the initial EPSC before triggering, *I*_1_ indicates the peak EPSC during triggering, and *I*_2_ indicates EPSC for 200 ms after triggering.

As shown in Figure S6, Supporting Information, the dextran‐OST showed an increasing response current (the ReC increased from 3.8 × 10^−4^ to 3.07 μA) with an increasing triggering voltage (from 0.5 to 3 V). Due to the accumulation effect of protons, the EDL does not immediately disappear after the removement of *V*_G_ pulses, resulting the long‐term plasticity (LTP). Therefore, the MeC was observed. And also, it showed an increasing memory current (the MeC increased from 7.5 × 10^−5^ to 0.409 μA) with an increasing triggering voltage (from 0.5 to 3 V). The memory current degree of the dextran‐OST increased from 0.18 to 1044 when the triggering voltage increased from 0.5 to 3 V. Similarly, the ReC, MeC, and ReD triggered by pulse width (from 100 to 500 ms) increased from 0.08 to 0.43 μA, 0.0013 to 0.042, and 2.57 to 83 μA, respectively. The results indicate that the voltage stimulation is much more efficient than the pulse width stimulation for the dextran‐OSTs.

The PPF index, which is defined as the ratio of the height of the second peak EPSC (*A*_2_) to the height of the first peak EPSC (*A*_1_) under the stimulation of two consecutive *V*_G_ pulses with the same shape, can be calculated by Equation (4).^[^ ^15^ ^]^

(4) $$\text{ PPF index}=\frac{A_{2}}{A_{1}}\times 100\%$$

A PPF index of as high as 435% was attained for the dextran‐OST when applying a pair of pulses with a width of 100 ms and an interval time (Δ*t*) of 100 ms (see the inset of Figure 2d). Figure S7, Supporting Information, shows the EPSCs of the dextran‐OST stimulated by two successive light pulses with different Δ*t* (50–500 ms). As depicted in Figure 2d, the highest PPF index value of 494% was obtained with a minimum interval of ≈50 ms, which is one of the best records compared with previous work (Table S1, Supporting Information).^[^ ^22^, ^23^, ^24^, ^25^, ^26^, ^27^, ^28^, ^29^, ^30^, ^31^, ^32^, ^33^ ^]^ With the time interval increases,^[^ ^34^ ^]^ electrons have enough time to recombine, so the PPF index will gradually decrease, and its variation can be fitted with the following Equation (5).^[^ ^35^ ^]^

(5) $$\text{PPF index}=C_{0}+C_{1}\times \exp\left(-\frac{\Delta t}{\tau_{1}}\right)+C_{2}\times \exp\left(-\frac{\Delta t}{\tau_{2}}\right)$$

where Δ*t* is the pulse interval, *C*_0_ is a constant, *C*_1_ and *C*_2_ are the initial facilitation amplitudes of the respective phases, and *τ*_1_ and *τ*_2_ are the relaxation time of the respective phases. The parameters extracted from the fitting curve are as follows (Figure 2d): *C*_0_ = 73%, *C*_1_ = 264%, *C*_2_ = 350%, *τ*_1_ = 126 ms, and *τ*_2_ = 180 ms. The relaxation time scales of the two phases are similar to those of a biological synapse. Figure 2e shows the change of EPSC with frequency when applying 10 consecutive pulses with different frequencies (1, 2, 3, and 4 Hz, corresponding to Δ*t* of 800, 300, 133, and 50 ms, respectively). The corresponding *A*_10_/*A*_1_ ratios are shown in Figure 2f (*A*_10_ is the height of the tenth peak EPSC). The *A*_10_/*A*_1_ ratios increased from 1.2 (1 Hz) to 3.1 (4 Hz), showing the typical filtering effect.^[^ ^36^, ^37^ ^]^ Figure 2g shows the potentiation and depression (PD) response of dextran‐OSTs under 10 PD cycles. Each stage includes 20 high‐level gate pulses (triggering voltage level of 2 V, resting voltage level of 1.5 V, pulse width of 100 ms) and 20 low‐level gate pulses (triggering voltage level of 1 V, resting voltage level of 1.5 V, pulse width of 100 ms), respectively. In each cycle, the EPSC increased after 20 potentiation pulses and then decreased after 20 depression pulses. Linearity is a crucial aspect of achieving accurate neuromorphic computations. To assess the linearity in relation to enhancement and suppression of the dextran‐OSTs, the parameter of nonlinearity (NL) was introduced. NL is defined as Equation (6).^[^ ^38^ ^]^

(6) $$\text{NL}=\text{average}\left(\left|\frac{I-\;I_{\text{Liner}}}{I_{\text{Liner}}}\right|\times 100\%\right)$$

where *I* represent the actual current and *I*_Linear_ represents the ideal linear current. The lower NL value means the higher linearity. As shown in Figure 2h, the dextran‐OST exhibits a nonlinearity of potentiation (NL_P_) of 2.63% and nonlinearity of depression (NL_D_) of −3.03%, respectively, showcasing exceptional linearity, consistency, and reproducibility. The dextran‐OSTs were used for image recognition by using the MNIST dataset to simulate a neural network in recognition tasks. The fully connected layers include 784 input neurons, 200 hidden neurons, and 10 output neurons, and the backpropagation algorithm was used for training. Figure 2i shows the schematic diagram of a representative MNIST handwritten digit “8” in a neural network and the recognition accuracy of the dextran‐OSTs. After 40 training periods, the recognition accuracy of the large (28 × 28 pixels) image reaches 89.95%. The simulation results demonstrate the potential of the dextran‐OST in image recognition.

### 2.3 Visual Synaptic Behaviors Regulated by UV‐Vis Light

**Figure 3:**
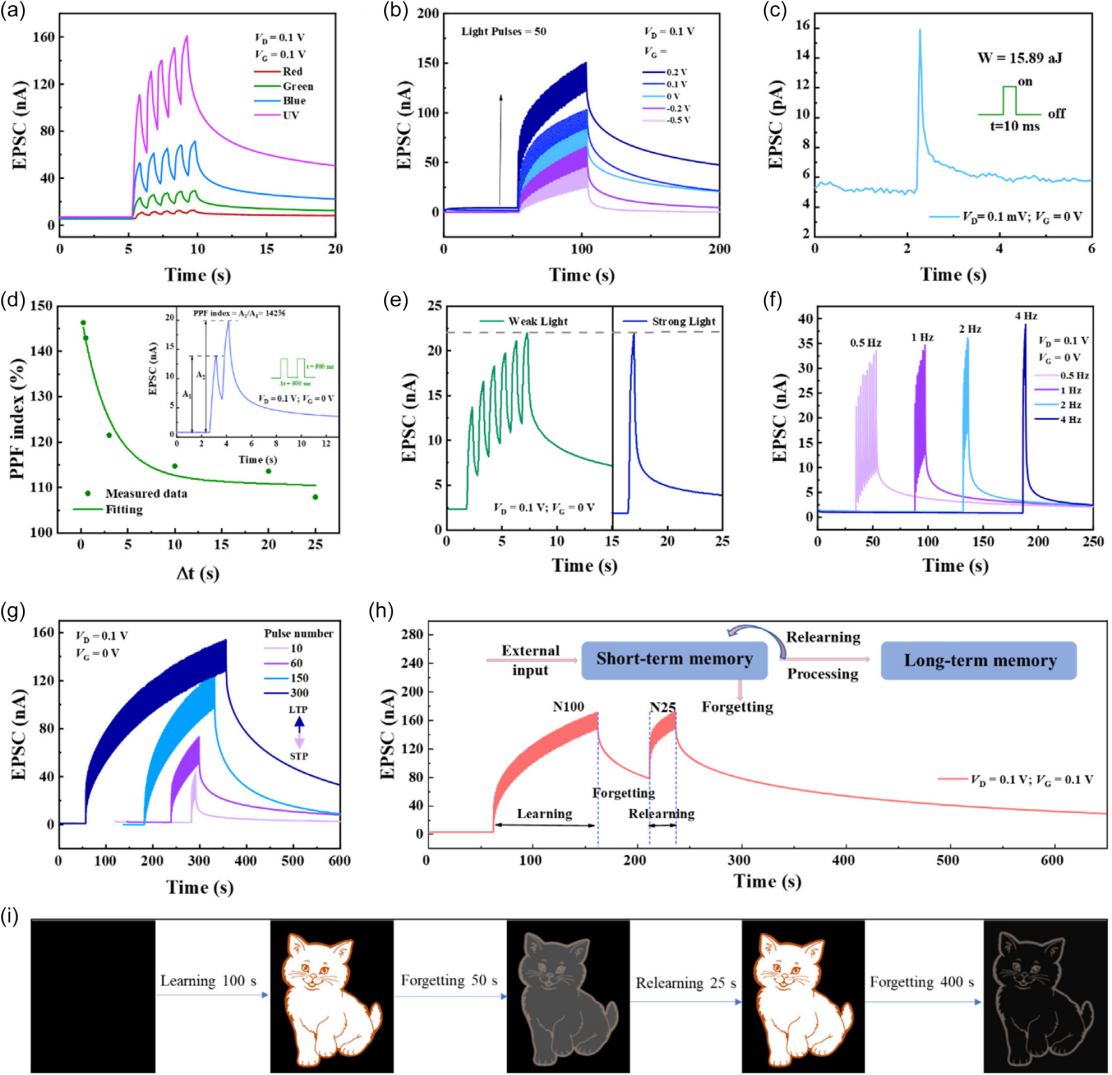
Synaptic performance under optical stimulation of the dextran‐OST. a) EPSC under different light wavelengths. Under white light stimulation: b) EPSC at different gate voltages. c) Energy consumption of a single neural activity. d) PPF index with different Δ*t* (inset: two successive presynaptic pulses with Δ*t* of 500 ms). e) EPSC equivalent to that of a strong stimulus by accumulating weak stimuli. f) Filter effect at different frequencies. g) Single presynaptic photo‐induced EPSC with different pulse numbers. h) Learning‐forgetting‐relearning characteristic. i) Simulated cat images to demonstrate distinct learning, forgetting and relearning behaviors.

Traditionally, artificial vision system with image processing and classification functions causes high power consumption and high latency for data transmission because of the separation of optical sensors, memories, and signal processing modules. In contrast, the retinal neurons in the human visual system (Figure 1a) not only detect light signals but also preprocess visual information, so the subsequent recognition and decision‐making tasks can be done more efficiently. The absorption spectrum of IGZO is shown in Figure S8a, Supporting Information, which exhibits that the dextran‐OSTs have the ability to realize broadband response from ultraviolet (UV) to visible light. Although the bandgap (*E*_G_) of IGZO is as wide as 3.3 eV (Figure S8b, Supporting Information). To investigate the light plasticity of the dextran‐OSTs, different light pulses with wavelengths ranging from UV (395 nm) to visible (blue: 465 nm, green: 520 nm, and red: 620 nm) were used for regulating the EPSC at a fixed optical power density, as shown in **Figure** **3**a. The dextran‐OST triggered by 5 successive UV light pulses had the highest EPSC, while the one triggered by visible light had lower EPSC (EPSC for blue > green > red). It is known that the existence of high‐density oxygen‐vacancy‐related subgap states (10^19^–10^20^ cm^−3^) near the valence‐band maximum (VBM) makes the IGZO semiconductor sensitive to subgap light (below ≈2.3 eV).^[^ ^16^ ^]^ Consequently, the optical response current of the IGZO semiconductor exhibits a dependence on wavelength. When applying light with longer wavelength (red and green) to the IGZO, the probability of the photoexcitation decreases, resulting in lower EPSC of the dextran‐OSTs. When applying light with a shorter wavelength (blue) to the IGZO, the probability of the photoexcitation increases, resulting in higher EPSC of the dextran‐OSTs. When applying UV light to the IGZO, the electrons can be excited directly from the valance band to the conduction band of IGZO, exhibiting a more significant EPSC. The response current (ReC), the memory current (MeC), and the memory degree (MeD) are shown in Figure S9, Supporting Information. It shows that shorter wavelength of light causes higher response and memory current. To further simulate the visual synaptic behaviors and the visual self‐adaptation of the dextran‐OSTs in a natural environment, white light (30 μW cm^−2^) was used to stimulate the dextran‐OSTs. The white light is a composite light range from 390 to 780 nm. Under 50 light pulses, the EPSCs can be enhanced or inhibited by *V*_G_ (Figure 3b). The principle of *V*_G_ regulation under light pulses is shown in Figure S10, Supporting Information. When *V*_G_ > 0 V, protons (H^+^) will accumulate at the interface of the channel/dielectric layer, which will stimulate more oxygen vacancies to participate in conduction and eventually increase the EPSC. When *V*_G_ < 0 V, oxygen vacancies in the channel are partially repaired, resulting in decreased EPSC. Actually, the EPSC of the dextran‐OSTs still exists when *V*_G_ = 0 V (Figure 3b). This may be attributed to the excellent PPC effect of IGZO (a large number of oxygen vacancies would still contribute to EPSC even when *V*_G_ = 0 V).

Energy efficiency is necessary for neuromorphic computation. When stimulated by a single light pulse, the EPSC signal (with an amplitude of 16 pA) remains clearly detectable even at a *V*_D_ of 0.1 mV. According to Equation (7)^[^ ^15^ ^]^

(7) $$W=V_{D}\times I_{D}\times t$$

the minimum energy consumption of the dextran‐OST single‐event operation was 15.89 aJ (Figure 3c), which is much lower than most of the previous works (Table S2, Supporting Information).^[^ ^23^, ^39^, ^40^, ^41^, ^42^, ^43^, ^44^, ^45^, ^46^, ^47^ ^]^ Figure 3d shows the PPF index of the dextran‐OST stimulated by two successive white light pulses with different Δ*t*. The PPF index under light pulse with Δ*t* of 500 ms is 142%. The parameters extracted from the curve fitted by Equation (5) are as follows: *C*_0_ = 60%, *C*_1_ = 36%, *C*_2_ = 51%, *τ*_1_ = 3 ms, and *τ*_2_ = 832 ms. The PPF indexes under different Δ*t* (from 250 ms to 25 s) are shown in Figure S11, Supporting Information.

In human visual system, the pupil of the human eyes will dilate to get more light in a dark environment with weak light, which is called visual adaptation. This dark adaptation behavior (a kind of visual adaptation behavior) suggests that the human retina can multiply and accumulate weak light. Figure 3e shows the visual adaptation behavior of the dextran‐OST. The dextran‐OST stimulated by 6 consecutive low‐intensity (8 μW cm^−2^) light pulses reached the same EPSC level as that stimulated by a high‐intensity (30 μW cm^−2^) light pulse. Besides, flicker fusion is another typical visual adaptation function in that the human retina will perceive a discrete segment of light as continuous when the frequency of the light is increased to a certain level.^[^ ^48^ ^]^ This suggested that the human retina plays a role similar to that of a low‐pass filter, which preprocesses and stabilizes fast signals thus preventing visual fatigue caused by high‐frequency signals. The simulation of the flicker fusion effect enables the dextran‐OST to filter out high‐frequency signals and retain low‐frequency signals in artificial vision systems (Figure 3f).

Longer memory can be formed by repeating memorization cycles or by extending the memory time, which can be emulated by adjusting the pulse number and pulse width, respectively. With the increase of the pulse number (10–300, Figure 3g) and pulse width (from 10 to 30 s, Figure S12, Supporting Information), the EPSC increased, and it took more time to recover to the original state, which indicates the conversion from STP to LTP. “Learning‐forgetting‐relearning” behavior is a critical indicator of the learning and memory ability of human brains.^[^ ^6^ ^]^ As shown in Figure 3h, after a period of learning by applying 100 successive light pulses, the EPSC increased to a peak value and slowly decreased. After 50 s forgetting, the EPSC was still much larger than the initial EPSC, which reflects that the dextran‐OSTs have good memory ability. After the second learning period with much fewer pulses (25 successive pulses), the dextran‐OST reached a higher EPSC than that after the first period of learning strength, indicating that the dextran‐OSTs have certain associating and relearning abilities. Moreover, after the second learning period, the forgetting time increased to 400 s. This indicates that the dextran‐OSTs can reduce the learning time and increase the memory time through training. The result shows that the dextran‐OSTs have great potential for deep learning ability.

The changes of the normalized EPSC in Figure 3i were mapped to the changes in brightness of a “cat” image. Firstly, the EPSC was normalized according to the maximum value of EPSC after being triggered by 100 pulses of light, and the normalized EPSC was recorded to form the original “cat” image (N100, Figure 3i). Then, the shape of the “cat” image (EPSC) was recorded again after the first period of 50 s forgetting (50 s after N100), the relearning period of 25 pulses of light (N25), and the second period of 400 s forgetting (400 s after N25). After the first period of 100 s forgetting, the image became obscure. After 25 pulses of relearning, the image became clear again. After the second period of 400 s forgetting (400 s after N25), the image became obscure again. The synaptic performance under 395, 465, 520, and 620 nm light stimulation of the dextran‐OSTs are shown in Figure S13–S16, Supporting Information, respectively. The higher PPF for the longer wavelength of 620 nm compared to those of 520 and 465 nm may be attributed to the different light absorption mechanisms. When stimulated by 520 and 465 nm light, the photoinduced electrons are mainly produced by ionization of oxygen vacancies (V_O_). For the 620 nm light, however, the photo energy of it is not enough to activate the V_O_, so the weak absorption of light may come from the oxygen adsorption–desorption effect on the surface of IGZO. The oxygen adsorption–desorption processes are chemical reactions which take a long time to react completely, while the V_O_ ionization is a photoelectric effect which take a relatively short time for relaxation. It results in a higher PPF under 620 nm light. It should be noted that the PPF under 395 nm (UV) light stimulation was higher than those under 465/520 nm light stimulation. It may be attributed to the trapping of the UV‐induced holes by the V_O_s. Because the photoenergy of the UV light is higher, hole–electron pairs can be generated under UV illumination. However, the holes will be trapped by the V_O_s because a large number of V_O_ states exist in the vicinity of VBM. It means that two processes take place under UV illumination. One is hole–electron pairs generation, and the other is hole trapping. As a result, the lifetime of the UV‐induced electrons is longer than those of the 465/520 nm light‐induced ones. This result demonstrates that the dextran‐OSTs not only perform excellent synaptic behaviors in the natural environment but also exhibit wavelength‐distinguished synaptic plasticity.

The EPSC responds differently to light and electricity mainly because of the different mechanisms between the electric double layer (EDL) effect and persistent photoconductivity (PPC) effect. When a positive electrical pulse is applied to the gate of the dextran‐OSTs, the H^+^ ions in the dextran film move to the dielectric/channel interface, forming EDL with large capacitance. Therefore, a large range of the EPSC can be obtained (Figure S17, Supporting Information). Unlike the electrical synaptic behavior, the light synaptic behavior of the dextran‐OSTs is mainly due to the PPC effect of the oxide semiconductor (IGZO), which can produce photoinduced electrons (Figure S18, Supporting Information).

### 2.4 Audio‐Visual Synergy Sensing

**Figure 4:**
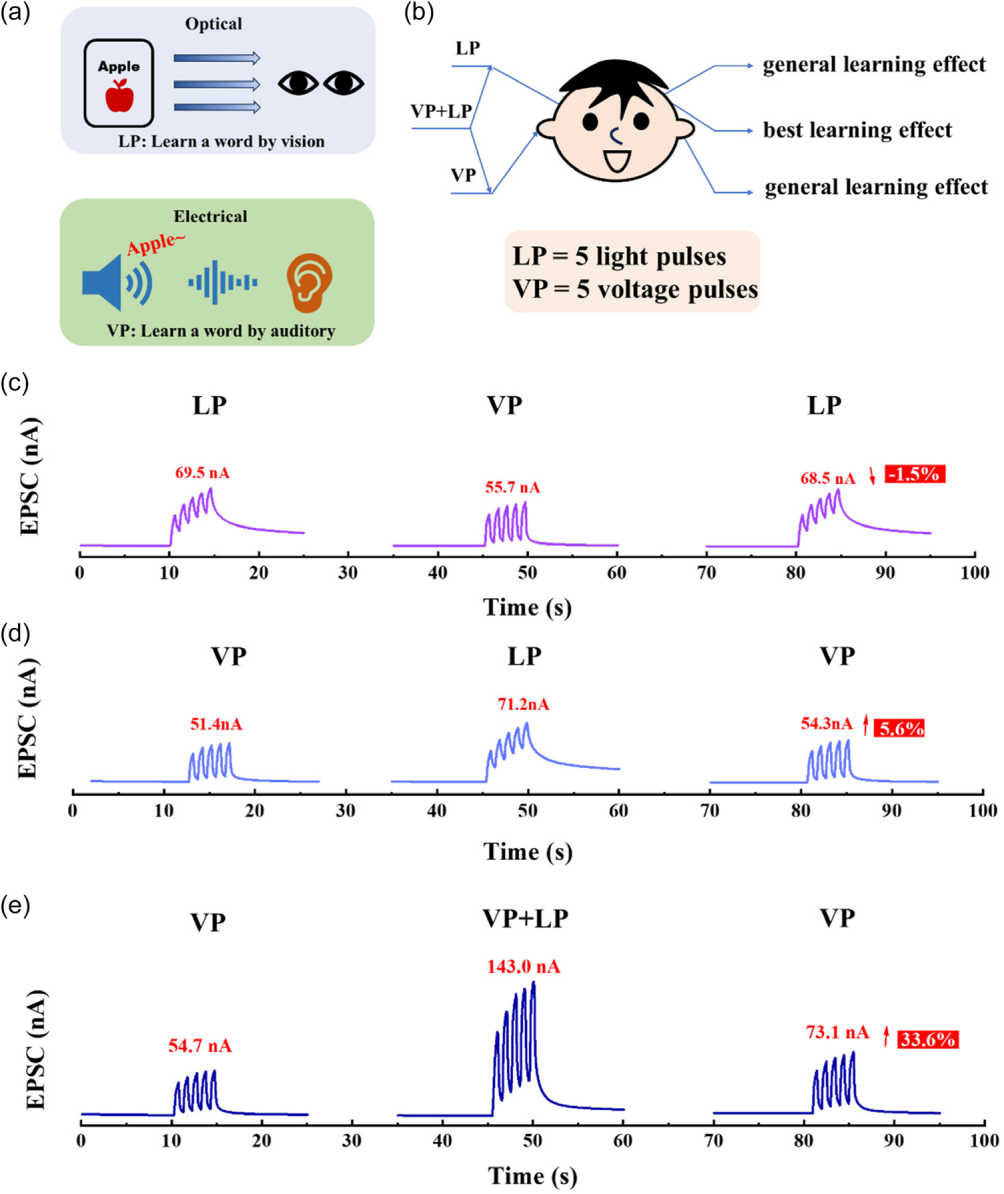
The audiovisual fusion effect of the optoelectronic synergy of the dextran‐OST. a) The action of light and electricity correspond to human visual and auditory sensations, respectively. b) The schematic diagram of better feedback under visual and auditory perception. c) The corresponding peak current variation of the LP‐VP‐LP processes. d) The corresponding peak current variation of the VP‐LP‐VP processes. e) The corresponding peak current variation of the VP‐(VP + LP)‐VP processes.

Visual memory and auditory memory are the basis of human memory.^[^ ^49^ ^]^ **Figure** **4**a,b shows a model of an infant learning the English word “apple,” where the flashcards with the spelling of a word represent learning and memory through vision, and the audio cues with the pronunciation and phonological structure of a word represent learning and memory through hearing. There are a variety of multimodal information presentation theories, such as Penney's modal effect separation flow hypothesis,^[^ ^48^ ^]^ Baddeley's working memory model,^[^ ^50^, ^51^ ^]^ and Mayer's cognitive theory of multimedia learning.^[^ ^52^ ^]^ According to modal learning effects, learning is enhanced when information is presented in both visual and auditory channels.^[^ ^49^ ^]^ When the two approaches are used simultaneously, the learning efficiency is significantly enhanced.

As shown in Figure 4c–e, the voltage (VP) and light (LP) pulses were used to simulate auditory and visual information, respectively. The *V*_D_ was set as 0.1 V, the triggering voltage level was 1 V, and the resting voltage level was 0.3 V. The white light power destiny was 30 μW cm^−2^. The obtained EPSC represents the learning level of the English words. When stimulated LP‐VP‐LP processes, the EPSCs of the dextran‐OST were 69.5, 55.7, and 68.5 nA for LP (1st stimulation), VP (2nd stimulation), and LP (3rd stimulation), respectively, a slightly 1.5% EPSC degradation between 1st and 3rd stimulations (Figure 4c). Applying a series of VP to the gate of the dextran‐OST is similar to applying positive/negative bias stress (PBS/NBS) to the gate of the dextran‐OSTs. The PBS will cause a positive shift of the threshold voltage (*V*_T_), and the NBS will cause almost no *V*_T_ shift.^[^ ^53^ ^]^ When stimulated by LP‐VP‐LP processes, the LP‐induced electrons will be recombined more rapidly by the following VP (PBS/NBS) process, and some of the electrons will be trapped by the positive period of the VP. As a result, less photoelectrons will be induced by the last LP process. When stimulated VP‐LP‐VP processes, the EPSCs of the dextran‐OST were 51.4, 71.2, and 54.3 nA for VP (1st stimulation), LP (2nd stimulation), and VP (3rd stimulation), respectively, a slightly 5.6% EPSC enhancement between 1st and 3rd stimulations (Figure 4d). In this process, the second step of LP process will induce electrons, some of which will be reserved in the following VP (PBS/NBS) process. As a result, more electrons will accumulate at the IGZO/dextran interface during the last VP process. The result indicates that the brain's processing of visual and auditory information is relatively independent. Based on the modality‐switch effect, when two different modalities of information alternate, the brain's reactions have additional performance costs in terms of speed and accuracy.^[^ ^54^, ^55^ ^]^ Therefore, the simple modality switch cannot enhance the learning process (or even has a negative effect on the learning process). However, when the VP and LP were input simultaneously (VP + LP) to simulate relevant auditory and visual information, the learning ability can be enhanced greatly. Figure 4e shows the EPSCs of the dextran‐OST stimulated with VP‐(VP + LP)‐VP processes. The EPSCs of the dextran‐OST were 54.7, 143.0, and 73.1 nA for LP (1st stimulation), VP + LP (2^nd^ stimulation), and LP (3^rd^ stimulation), respectively, with a great EPSC enhancement of 33.6% between 1st and 3rd stimulations. In this process, the second step of VP + LP process will induce a large number of electrons (similar to PBIS/NBIS process). As a result, much more electrons will accumulate at the IGZO/dextran interface during the last VP process. This indicates that receiving visual and auditory information simultaneously leads to a deeper level of learning and memory.

Figure S19, Supporting Information, shows a schematic diagram of the classical conditioning training process for the Pavlov's dog. The *V*_D_ was set as 0.1 V, the triggering voltage level was 0.35 V, and the resting voltage level was 0 V. The white light power destiny was 30 μW cm^−2^. The light signals were regarded as a feeding food (unconditioned stimulus) and the electrical signal was considered as a ringing bell (conditioned stimulus). Assuming that a current threshold of 50 nA is required for the dog to produce saliva, single electrical pulse cannot make the dog secrete saliva, showing that conditioned stimulus cannot function without training. However, when the voltage and light signals were synchronously applied as the training modules for dogs. After one training course, a single voltage pulse stimulation exceeded the threshold for dogs to secrete saliva, and further training can reach higher EPSC. It means multiple training courses can improve the learning response of associative learning. The results indicated that dextran‐OST establishes a connection between electrical and optical pulses, which is particularly advantageous for the application in human brain associative learning systems.

### 2.5 Applications in Optoelectronic Reservoir Computing

**Figure 5:**
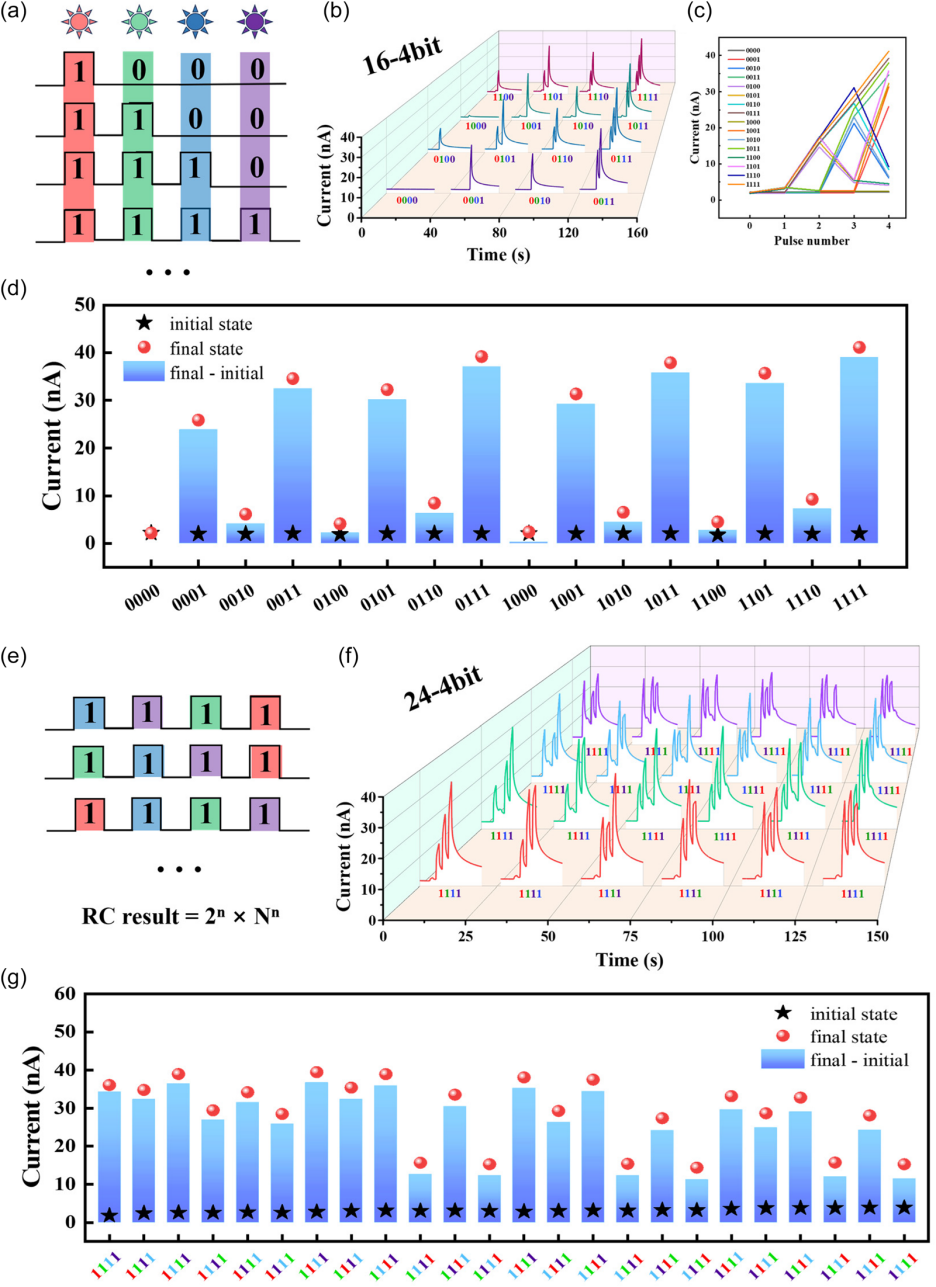
Nonlinear mapping of multimodal signals based on the dextran‐OST reservoirs for optical binary communication. a) Combination mode for 16 inputs of 4‐bit multiband optical pulses. b) Response current induced by 4‐colors (fixed order). c) The distinguishable output of 16 inputs of 4‐bit multiband optical pulses. d) Initial and final current values of the reservoir state with pulse stimulation of 16 combinations in the 16 inputs mode. e) Combination mode for 24 inputs of 4‐bit multiband optical pulses. f) Response current induced by 4 colors (not fixed in order). g) Initial and final current values of the reservoir state with pulse stimulation of 24 combinations in the 24 inputs mode.

In order to evaluate the multimodal sensing capability of optoelectronic reservoirs, 4‐bit binary streams of different wavelengths (including red (R), green (G), blue (B), and ultraviolet (UV)) fused input modes were applied. As shown in **Figure** **5**a,b, each square wave input is considered as one bit and the 4‐bit input stream was encoded as a pulse sequence from “0000” to “1111,” in which the “off” and “on” state of the optical pulse denote “0” and “1”, respectively. Figure 5c shows the temporal variation of the drain currents for 16 different inputs, indicating that the final state of the reservoir is not only dependent on the last stimulus but also related to the history of external stimuli. Figure 5c illustrates the evolution of the channel current after each pulse stimulation. Due to the nonlinear relaxation characteristics of the reservoir, the final state depends on its activity history. Therefore, “0001” and “0010” are two different sequences for the reservoir. The final states of 16‐bit can be distinguished from each other even with the same initial state (Figure 5d). When classified into 24 combinations, namely “R‐G‐B‐UV,” “R‐UV‐G‐B,” “G‐B‐R‐UV,” “G‐B‐UV‐R,” and so on (Figure 5e,f), the final states of 24 bit can also be distinguished from each other even with the same initial state (Figure 5g). The multiband dextran‐OSTs can increase the reservoir greatly, which means that there are 4,096 different combinations (*C*) when 4‐bit dextran‐OSTs are designed as dual network structures (*N* = 4, *n* = 4), as described in Equation (8)^[^ ^56^ ^]^

(8) $$C=2^{n}\times N^{n}$$

where *n* is the number of wavelengths and *N* is the number of pulses. The distinguishability of the *C* = 4096 states indicates the potential of the dextran‐OSTs for in‐memory RC applications.^[^ ^57^ ^]^

## 3 Conclusions

In summary, a novel dextran‐OST with multimodal neuromorphic computation and retinal‐inspired multiband optical binary communication is successfully demonstrated. The dextran‐OSTs exhibit excellent electrical performance with a high mobility of 14.3 cm^2^ V^−1^ s^−1^. To construct electrical/optical multimodal neuromorphic computation, a wide range of optoelectronic synaptic characteristics have also been emulated, including STP/LTP, a high PPF index of 494%, SVDP/SFDP/SNDP/SDDP, learning‐forgetting‐relearning feature, and a high recognition accuracy of 89.95% by handwritten digital datasets. Besides, visual adaptation and audiovisual fusion effects were simulated, which proved that the dextran‐OSTs had visual self‐adaptation and synergy sensing abilities. In addition, the optical communication based on multiband binary reservoir computing (*λ* = 620, 520, 465, and 395 nm) was successfully constructed. Unlike the current devices that respond to different light sources separately, the dextran‐OSTs can discriminate light of different light wavelengths when switching different light wavelengths and achieve retina‐inspired multiband optical binary communication (retina‐inspired intelligent communication). This research promotes the development of emerging brain/retina‐inspired bionic devices that have the characteristics of multimodal interaction and optical information dissemination.

## 4 Experimental Section

4.1

4.1.1

##### Preparation of Dextran Precursor

The dextran precursor solution was prepared by adding 60 mg dextran powder (*M*_w_ = 500 000, Shanghai Fusheng Industrial Co. Ltd.) to 940 mg deionized water, heating to 70 °C, and stirring for 12 h.

##### Device Fabrication

Figure S1, Supporting Information, shows the fabrication process of the dextran‐OSTs. Firstly, a 150 nm‐thick ITO was deposited on glass substrates as a bottom gate electrode. Then, the dextran films were spin coated at a speed of 3500 rmp to form a 100 nm dextran film. Secondly, a layer of 20 nm IGZO film was deposited by direct–current (DC) magnetron through a shading mask and annealed at 80 °C for 1 h. Finally, the Al film (150 nm) was deposited by vacuum evaporation through another shading mask to form the source/drain electrodes (S/D).

##### Characterization

The UV‐vis absorption spectra (UV‐vis spectra) of the IGZO films were measured by a UV‐vis spectrophotometer (UV‐2600). The electrical characteristics and optical response of the phototransistors were examined in the lab under dark and illuminated conditions using a semiconductor parameter analyzer. During testing, the hold time and delay time were set to 500 and 200 ms, respectively. (Agilent B1500).

##### Calculation

The dextran‐OSTs work in the linear region, and the *I*_D_ in the linear region is expressed by the following empirical Equation (9)^[^ ^58^ ^]^

(9) $$I_{D}=\mu_{\text{linear}}C_{i}\frac{W}{L}\left(V_{G}-V_{T}-\frac{V_{D}}{2}\right)V_{D}$$

where *W* is the width of the channel, *L* is the length of the channel, *μ*_lin_ is the mobility of the dextran‐OSTs in the linear region, *C*_i_ is the unit capacitance of the insulating layer, and *V*_T_ is the threshold voltage of the device. As shown in Figure 1c and S20, Supporting Information, the *μ*_lin_ was calculated to be up to 14.3 cm^2^ V^−1^ s^−1^.

## Conflict of Interest

The authors declare no conflict of interest.

## Author Contributions

**Bo Huang**: conceptualization (lead); data curation (lead); formal analysis (lead); funding acquisition (lead); investigation (lead); methodology (lead); project administration (lead); resources (lead); software (lead); supervision (lead); validation (lead); visualization (lead); writing—original draft (lead); writing—review and editing (lead). **Linfeng Lan**: conceptualization (lead); data curation (lead); formal analysis (lead); funding acquisition (lead); investigation (lead); methodology (lead); project administration (lead); resources (lead); software (lead); supervision (lead); validation (lead); visualization (lead); writing—original draft (lead); writing—review and editing (lead). **Jiayi Pan**: conceptualization (lead); data curation (lead); formal analysis (lead); funding acquisition (lead); investigation (lead); methodology (lead); project administration (lead); resources (lead); software (lead); supervision (lead); validation (lead); visualization (lead); writing—original draft (lead); writing—review and editing (lead). **Fuzheng Qi**: data curation (equal); software (equal); supervision (equal); validation (equal). **Jing Li**: data curation (equal); formal analysis (equal); software (equal); validation (equal). **Churou Wang**: formal analysis (equal); funding acquisition (equal); software (equal); validation (equal). **Yaping Li**: data curation (equal); formal analysis (equal); resources (equal); validation (equal). **Dechun Zeng**: data curation (equal); funding acquisition (equal); supervision (equal); validation (equal). **Jiale Huang**: formal analysis (equal); funding acquisition (equal); software (equal); validation (equal). **Jintao Xu**: data curation (equal); formal analysis (equal); software (equal); validation (equal). **Junbiao Peng**: data curation (equal); formal analysis (equal); software (equal); validation (equal).

## Supporting information

Supplementary Material

## Data Availability

The data that support the findings of this study are available from the corresponding author upon reasonable request.
