# Supplementary material for: Low‐Power Optoelectronic Synaptic Transistors with Multimodal Neuromorphic Computation and Retinal‐Inspired Multiband Optical Binary Communication
Source: Small Sci. 2025 Jan 21;5(5):2400511. doi: 10.1002/smsc.202400511 (PMC12087769; doi:10.1002/smsc.202400511)
Supplement: Supplementary file 1 — Supplementary Material [file SMSC-5-2400511-s001.pdf]

Low-power optoelectronic synaptic transistors with multimodal neuromorphic computation and retinal-inspired multiband optical binary communication

Bo Huang, Linfeng Lan, Jiayi Pan, Fuzheng Qi, Jing Li, Churou Wang, Yaping Li, Dechun Zeng, Jiale Huang, Jintao Xu and Junbiao Peng*

B. Huang, Prof. L. F. Lan*, J. Y. Pan, F. Z. Qi, J. Li, C. R. Wang, Y. P. Li, D. C. Zeng, J. L. Huang, J. T. Xu and Prof. J. B. Peng

**Corresponding author: lanlinfeng@scut.edu.cn*

State Key Laboratory of Luminescent Materials and Devices
South China University of Technology
Wushan Road 381, Guangzhou 510640, P. R. China
E-mail: lanlinfeng@scut.edu.cn

MINST is widely used in image recognition of synaptic transistors. Due to the simplicity of the data set and the completeness of the pre-processing, it is well suited for machine learning and deep learning. In addition, there are a large number of open-source code, tutorials and research papers on the MNIST dataset, which provide a rich resource for image recognition research on synaptic transistors.

We chose Cross-sim simulator to simulate the device. The simulation method is as follows:

The Cross-sim simulator simulates the cyclic of potentiation and depression (PD). The number of simulations of the model and the number of cycles of each simulation can be set in the Cross-sim. MINST is a 28×28 pixel handwritten digital data set, so a three-layer neural network with input layer 784, intermediate layer 300 and output layer 10 is set in Cross-sim respectively for neural network simulation. There are a total of 70000 images in MINST, including 60000 training images and 10000 test images. In each cycle, 60000 training images will be input to train the simulated synaptic array. After the training, 10000 test images will be used for accuracy statistics.

In Cross-sim, the learning rate of the neural network can be adjusted. By setting a bias weight, the learning rate of the neural network can be adjusted through the training process to better fit the training data. Cross-sim can simulate the learning accuracy of theoretical device array simulation and single device array simulation. By comparing the accuracy of the two, we can observe whether the performance of the device is excellent in image recognition applications.

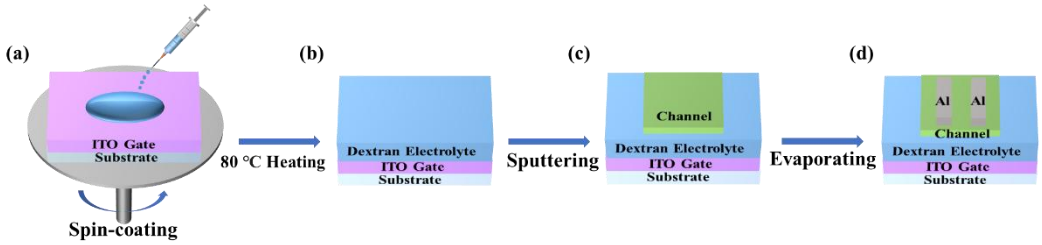

Figure S1. Fabrication scheme and electrical properties of the dextran-OSTs.

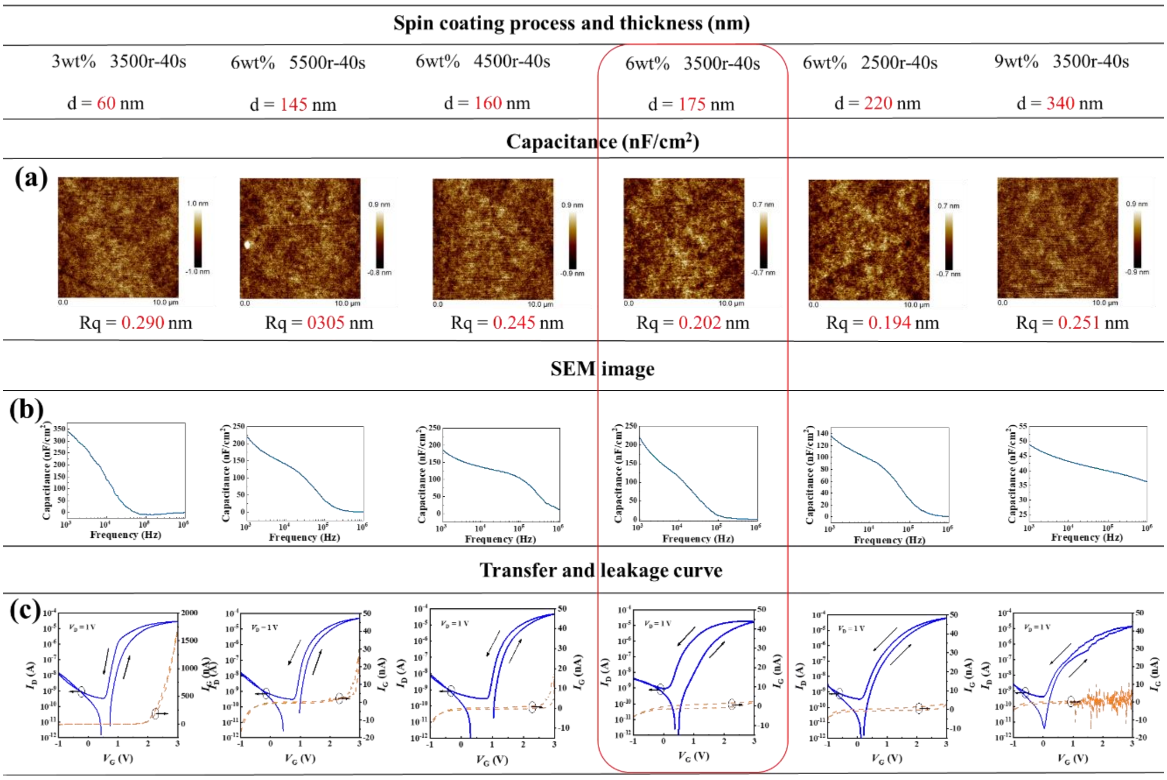

Figure S2. Properties of dextran thin films and electrical properties of dextran-OSTs under different spin coating processes. (a) SEM image (b) Capacitance frequency (C-F) curve. (c) transfer curve of the dextran-OSTs.

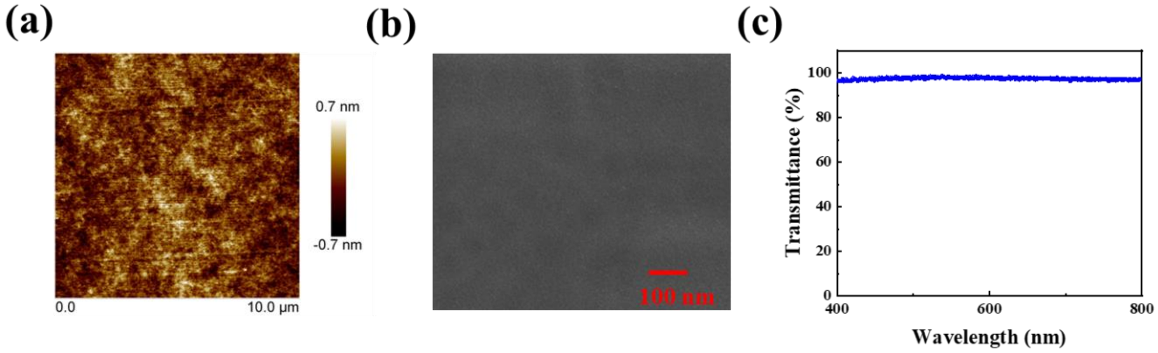

Figure S3. The characteristic of the optimized dextran film (a) AFM image of the dextran film. (b) SEM image of the dextran film. (c) Transmission spectrum curve of the dextran film.

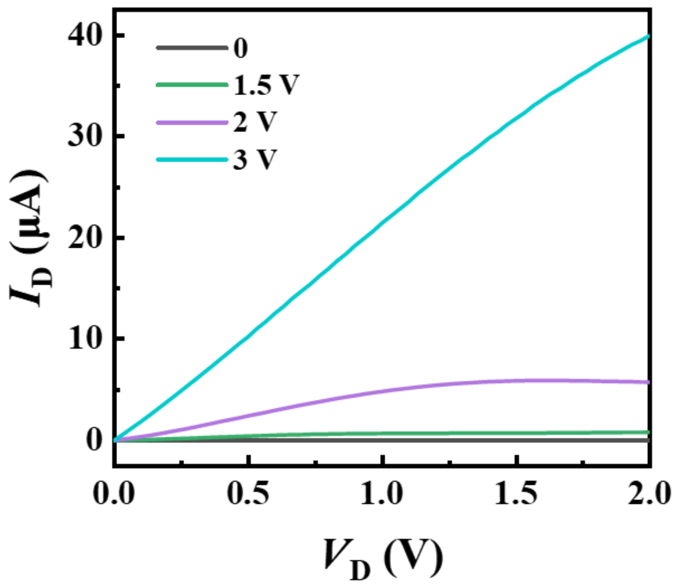

Figure S4. Output curve of the dextran-OST.

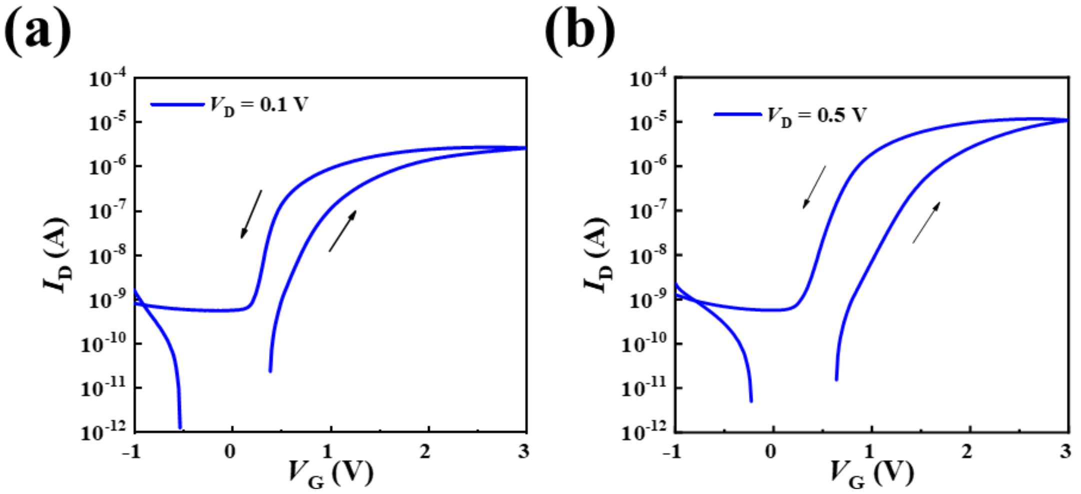

Figure S5. Transfer curve of the dextran-OSTs at (a) V_D = 0.1 V and (b) V_D = 0.5 V.

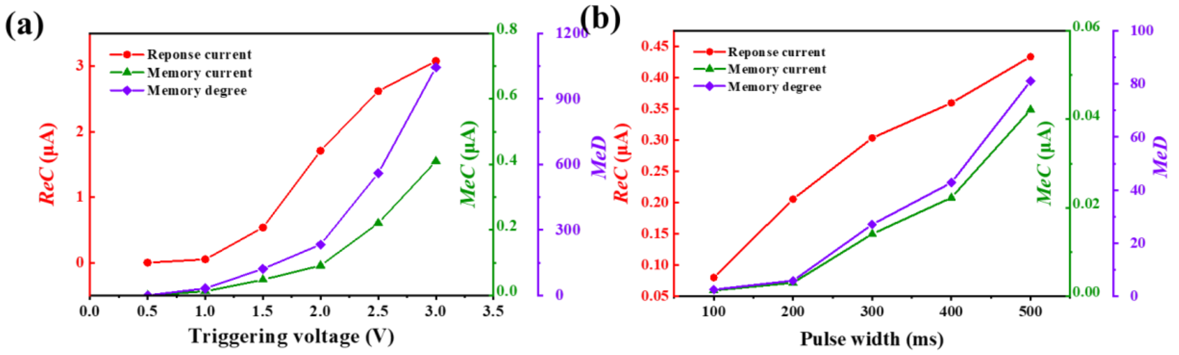

Figure S6. The response current (*ReC*), memory current (*MeC*) and memory degree (*MeD*) of the dextran-OSTs. The *ReC&MeC&ReD* (a) at different triggering voltage. (b) at different pulse width.

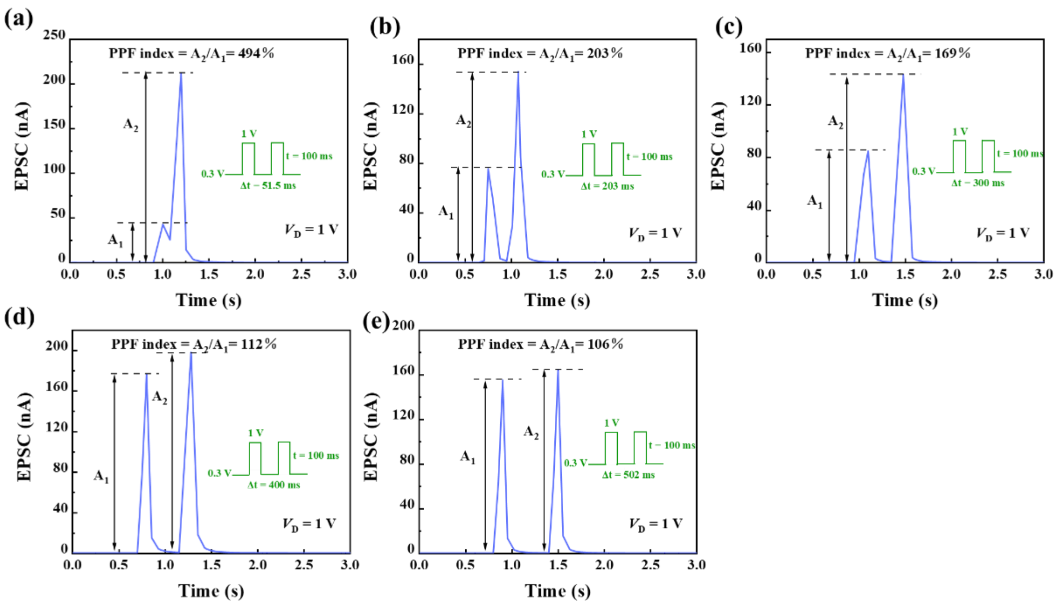

Figure S7. EPSC triggered by two successive electrical pulses with different *Δt***.** (a) 51.5 ms. (b) 203 ms. (c) 300 ms. (d) 400 ms. (e) 502 ms.

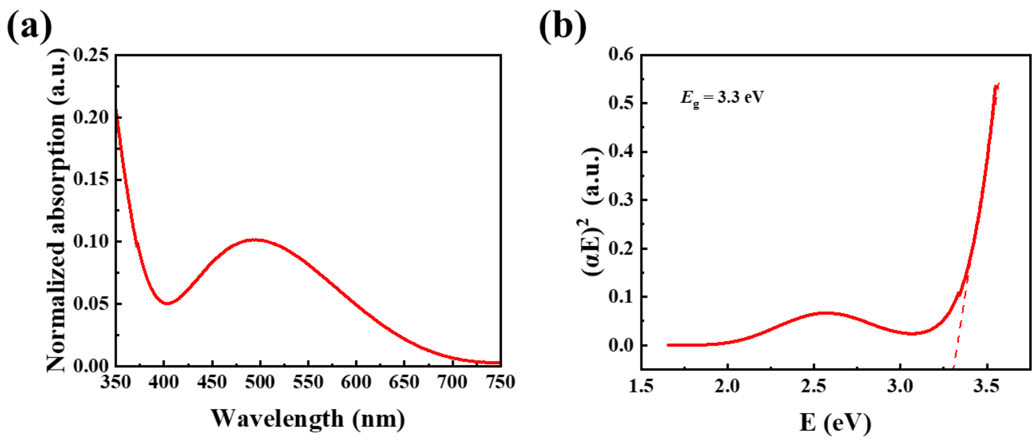

Figure S8. (a) The absorption spectrum of IGZO film. (b) The bandgap energy of IGZO was calculated as 3.3 eV.

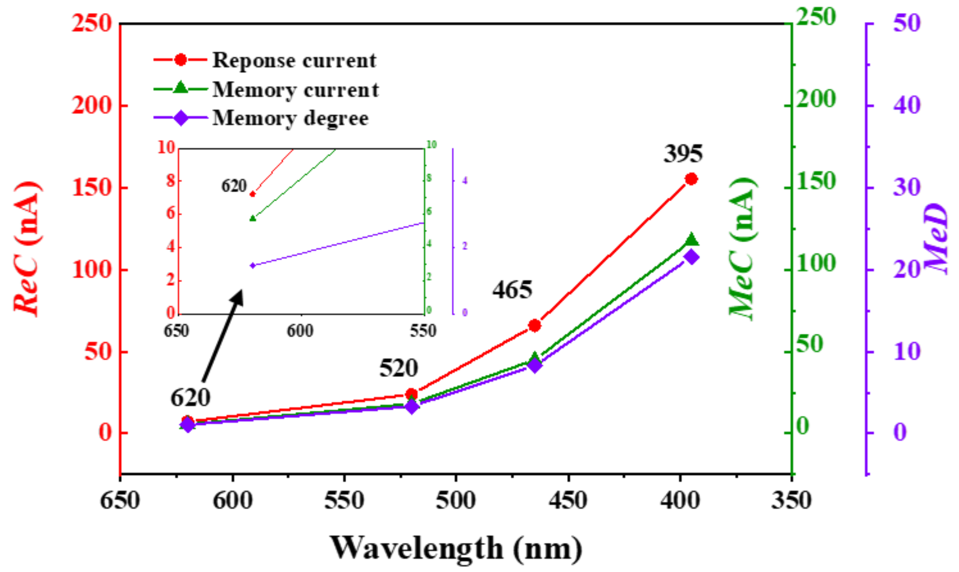

Figure S9. The response current (*ReC*), memory current (*MeC*) and memory degree (*MeD*) of the dextran-OSTs. *The ReC&MeC&ReD* at different light wavelength.

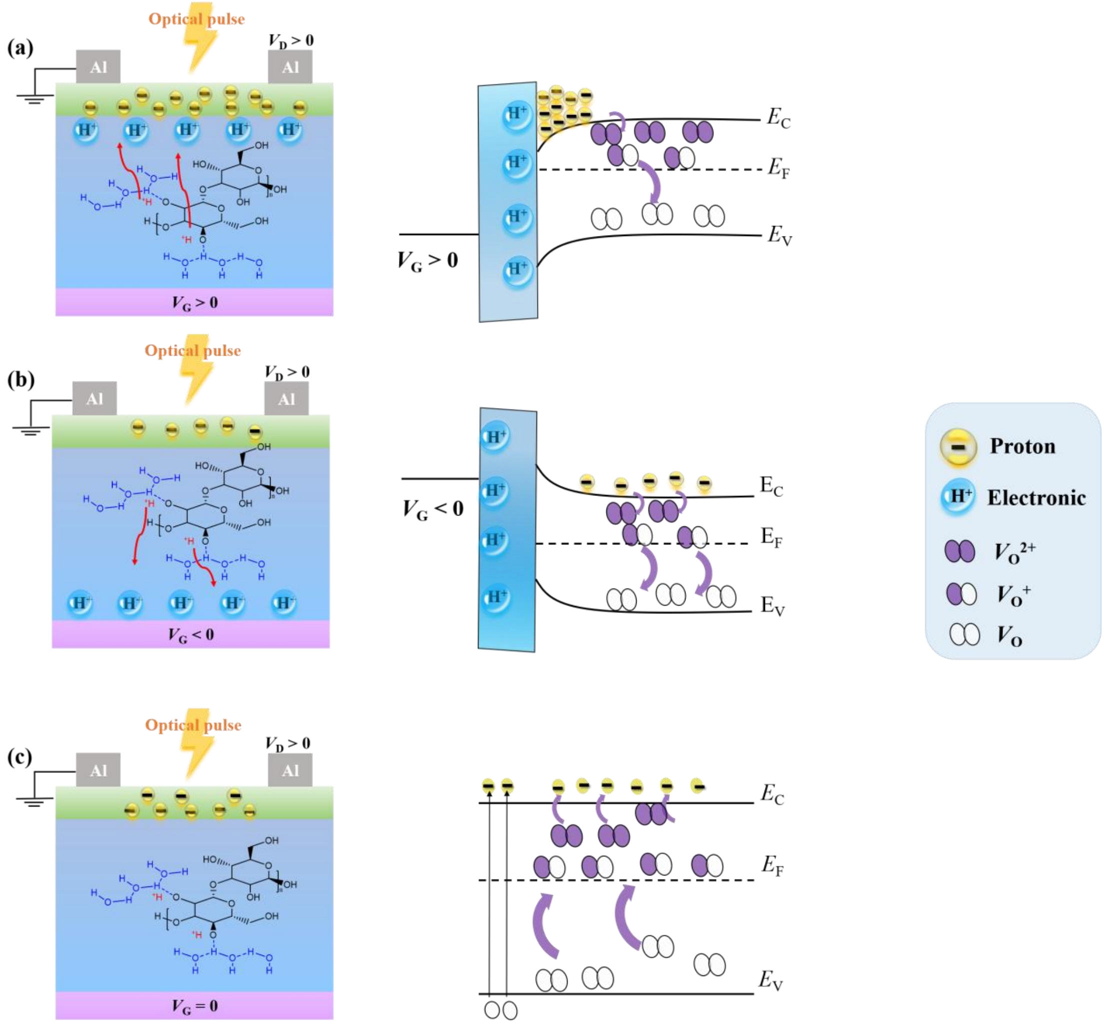

Figure S10. The structure and energy band diagram of dextran-OSTs. (a) $V_G > 0$ V. (b) $V_G < 0$ V. (c) $V_G = 0$ V.

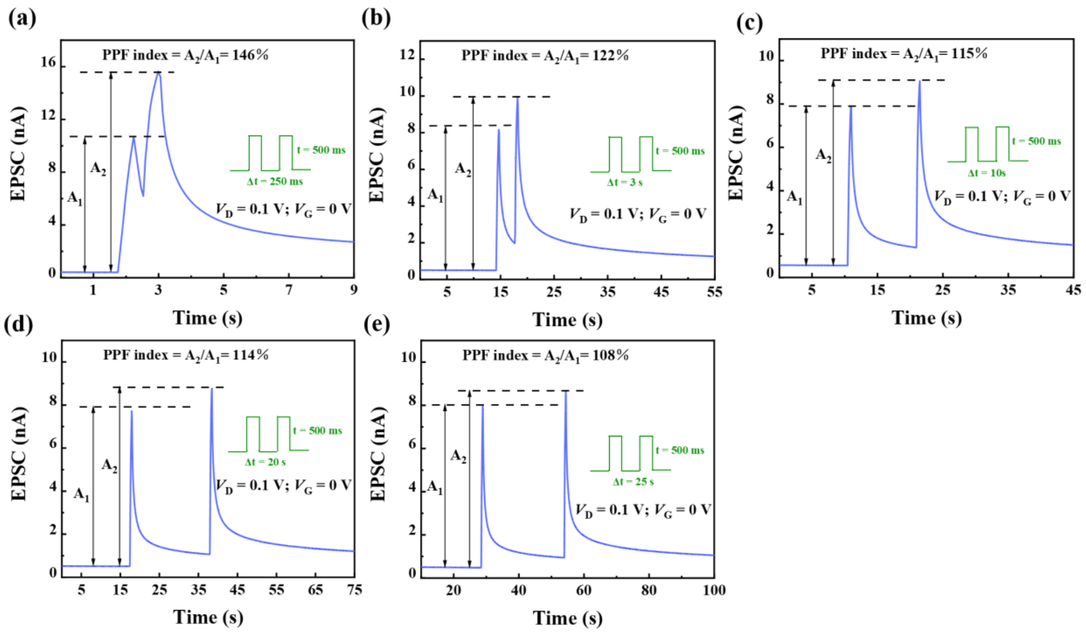

Figure S11. EPSC triggered by two successive light pulses with different *Δt*. (a) 250 ms. (b) 3 s. (c)10 s. (d) 20 s and (e) 25 s.

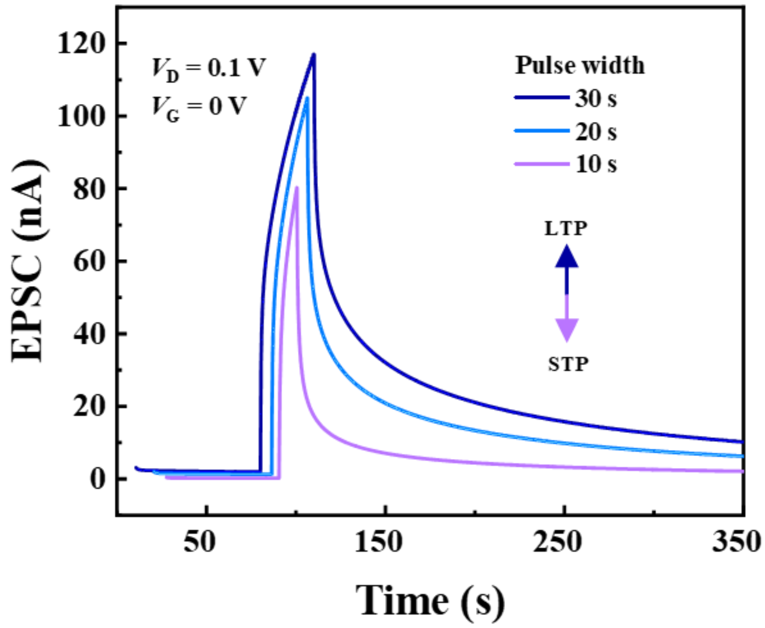

Figure S12. Synaptic plasticity of the dextran-OST stimulated by different pulses of light.

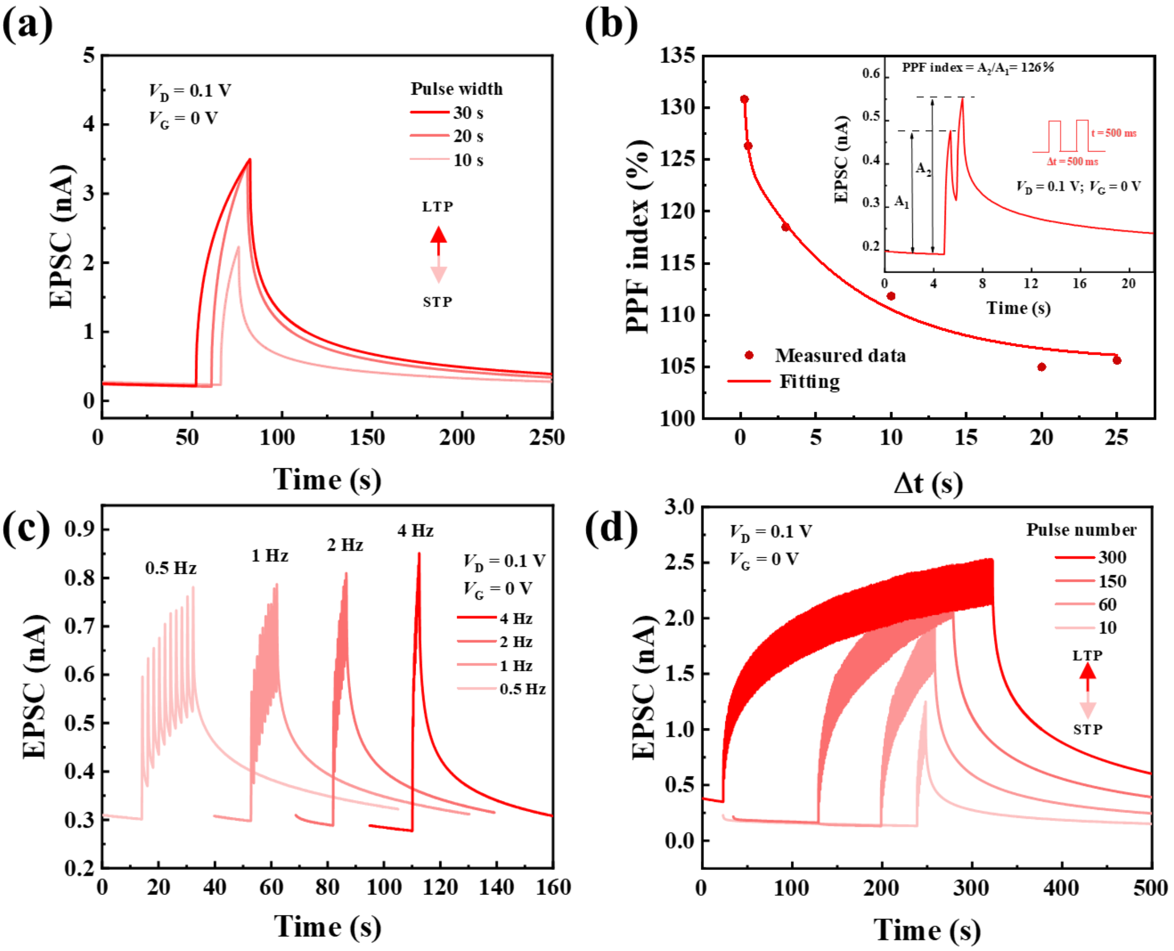

Figure S13. Synaptic performance under 620 nm light stimulation of the dextran-OST. (a) EPSC under different pulse width. (b) PPF index with different Δt (inset: two successive presynaptic pulses with Δt of 500 ms). (c) filter effect at different frequencies. (d) EPSC under different pulse numbers.

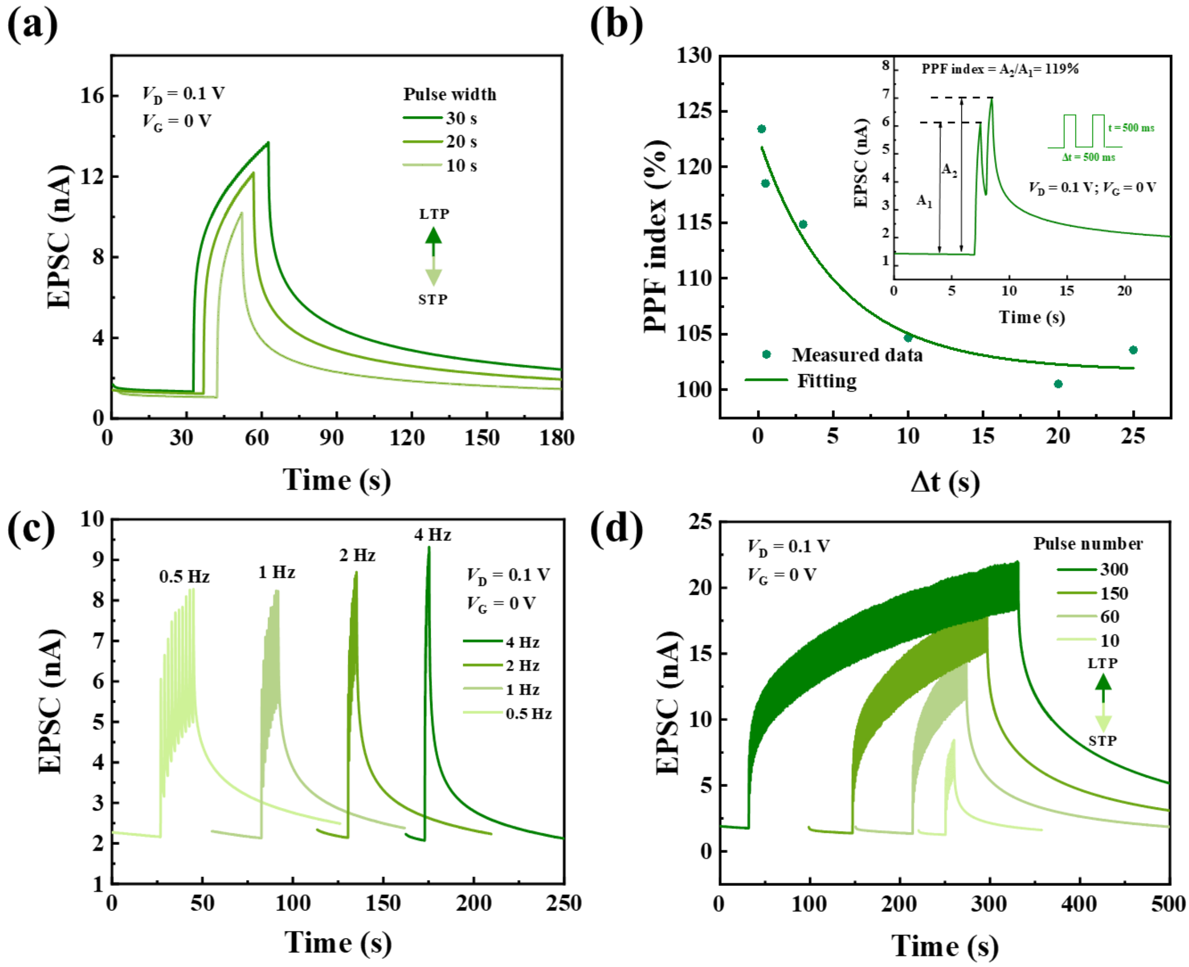

Figure S14. Synaptic performance under 520 nm light stimulation of the dextran-OST. (a) EPSC under different pulse width. (b) PPF index with different Δt (inset: two successive presynaptic pulses with Δt of 500 ms). (c) filter effect at different frequencies. (d) EPSC under different pulse numbers.

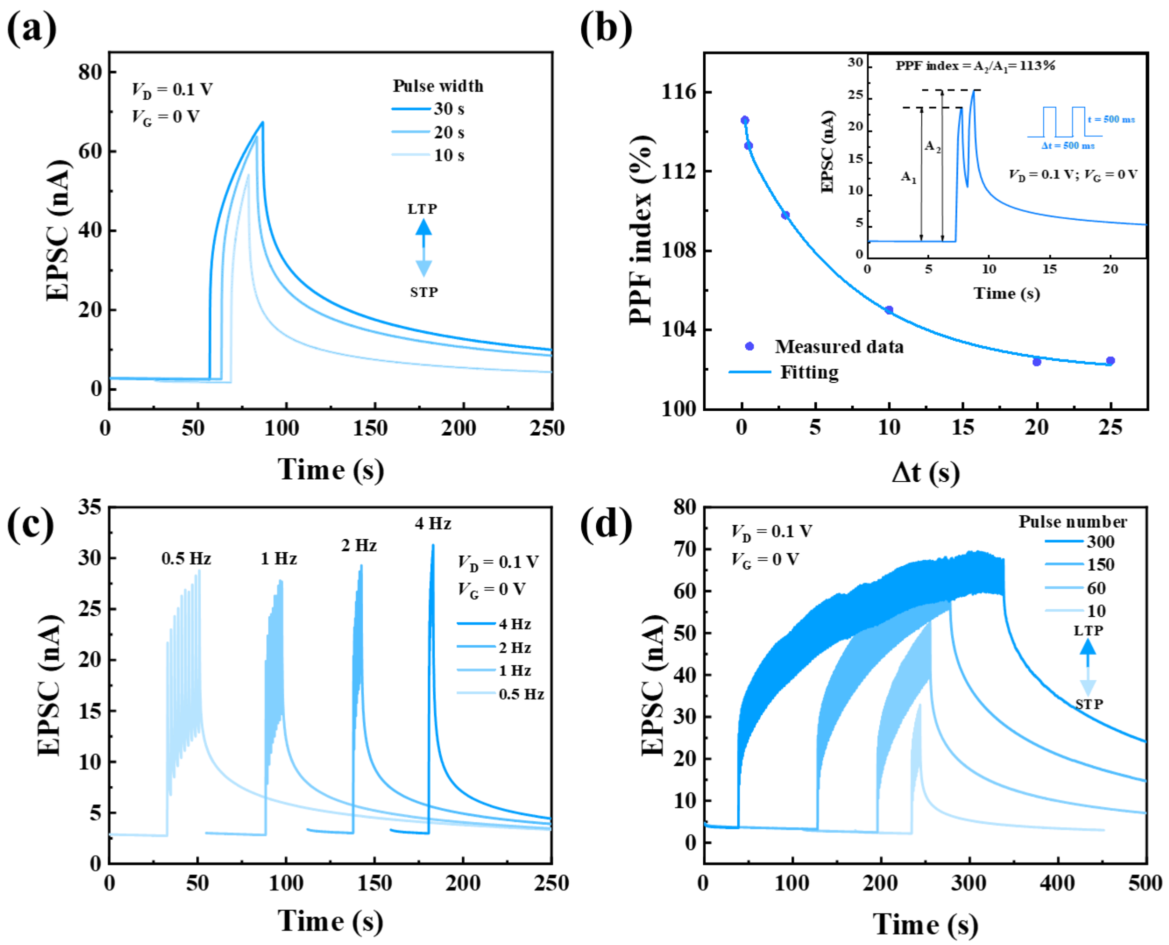

Figure S15. Synaptic performance under 465 nm light stimulation of the dextran-OST. (a) EPSC under different pulse width. (b) PPF index with different Δt (inset: two successive presynaptic pulses with Δt of 500 ms). (c) filter effect at different frequencies. (d) EPSC under different pulse numbers.

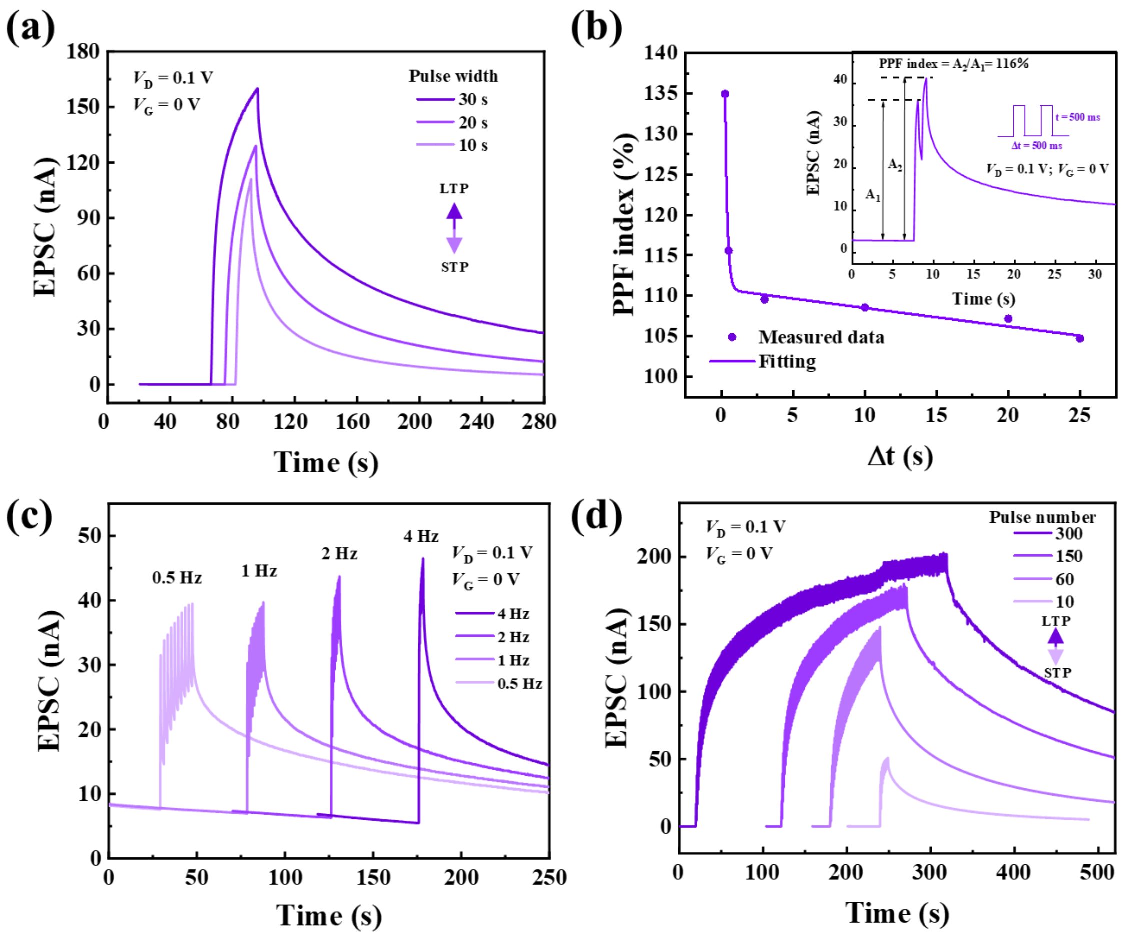

Figure S16. Synaptic performance under 395 nm light stimulation of the dextran-OST. (a) EPSC under different pulse width. (b) PPF index with different Δt (inset: two successive presynaptic pulses with Δt of 500 ms). (c) filter effect at different frequencies. (d) EPSC under different pulse numbers.

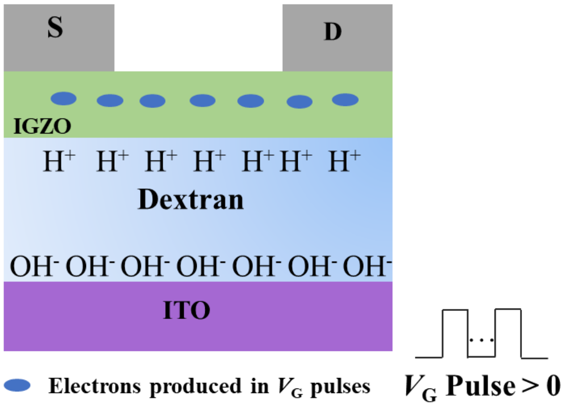

Figure S17. The EPSC stimulated under electrical pulses.

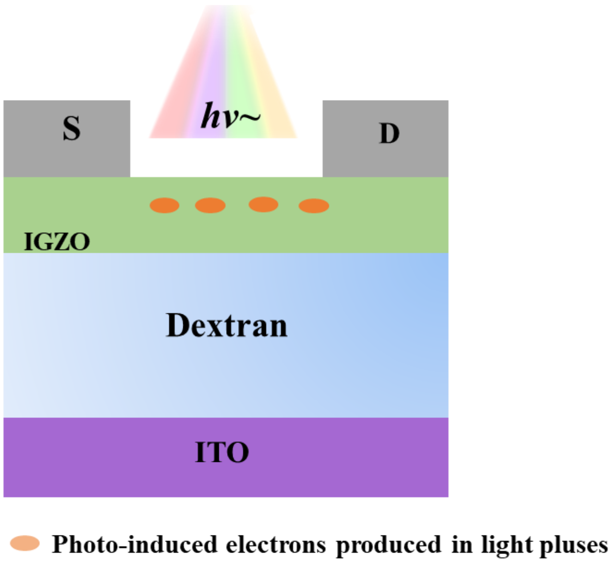

Figure S18. The EPSC stimulated under light pulses.

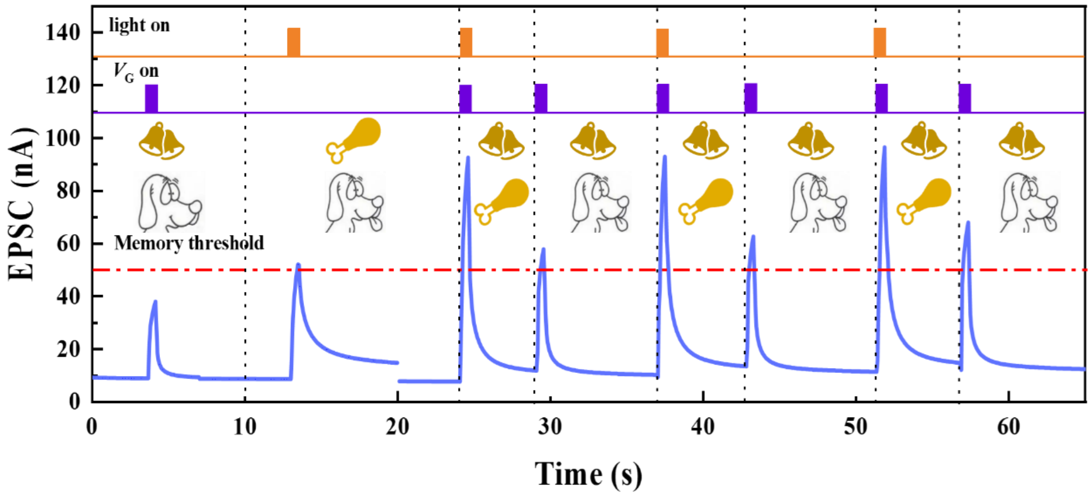

Figure S19. Pavlov's dog effect under optoelectronic synergy regulation. Electrical signal: as a ringing bell (conditioned stimulus); Light signal: as a feeding food (unconditioned stimulus); Training mode: voltage + light

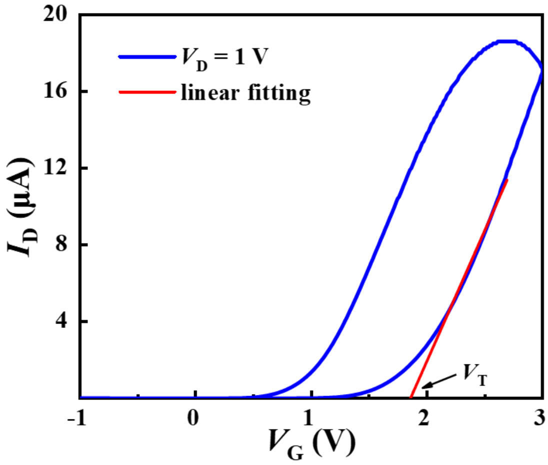

Figure S20. Transfer curve of the dextran-OSTs and the fitting result of V_T.

Table S1. Comparison of the PPF index among reported synaptic transistors under electrical or optical stimulation.

Material	Electrical PPF index	Δt (ms)	Optical PPF index	Δt (ms)	Ref.
**Al_2O_3+ITO**			195%*	200*	20
**Al_2O_3+InP/ZnSe QD/SnO2**			183%	100	21
**Al_2O_3+IO/IGZO**			268%*	100*	22
**SiO_2+IGZO:Cd300**			205%*	25*	23
**SiO_2+IGZO/m-TiO_2/IGZO/ m-TiO_2/HfO_2/IGZO**			721%	500	24
**SiO_2++PNCs /IGZO**			180%*	2000*	25
**SiO_2+IAZO**			155.9%	100	26
**SiO_2+PCz-sorted SWCNT**	467%	4600			27
**SiO_2+IZO**	195%*	20*			28
**ZrO_x-Li+ InO_x**	260%*	100*			29
**GO/Chitosan+IZO**	222%	30			30
**Chitosan+IGZO**	185%*	25*			31
**Dextran+IGZO**	494%	51.5	146%	250	This work

The “*” in table indicates that the specific numerical value was not published directly, and the data was estimated from the graph.

Table S2. Comparison of the energy consumption among reported synaptic transistors.

Semiconductor	Optical stimulation	Electrical stimulation	Energy consumption	Synaptic functions	Ref.
InP/ZnSe QD/SnO_2	✓	✓	~5.6 pJ	EPSC/LTP/STP/ LTD/ PPF	21
IGZO/$CsPbBr_3$-QDs	✓	✗	~30 pJ	EPSC/IPSC/LTP/STP	37
IZO	✓	✗	~0.35 nJ	EPSC/STP/LTP/ PPF	38
In_2O_3	✓	✓	~40 nJ	STP/LTP/STDP	39
MoS_2	✓	✓	~63 pJ	LTP/STDP	40
ITO/HfLaO	✗	✓	~93.1 aJ	STP/LTP/PPF	41
$CsPbBr_3$ QDs/PQT-12	✓	✗	~0.65 nJ	STP/LTP	42
TIPS-pentacene/PS/ CPBQDs	✓	✗	~0.036 fJ	EPSC/STP/LTP/ PPF/STDP/SNDP/ SFDP	43
sc-SWCNT	✓	✓	~11.3 nJ	EPSC/IPSC	44
sc-SWCNT	✓	✗	~15.38 aJ	EPSC/STP/STDP/SVDP	45
IGZO	✓	✓	~15.89 aJ	EPSC/STP/LTP/ PPF/SVDP/SFDP/SDDP/SNDP	This work
